# Interlayer‐State‐Coupling Dependent Ultrafast Charge Transfer in MoS_2_/WS_2_ Bilayers

**DOI:** 10.1002/advs.201700086

**Published:** 2017-04-24

**Authors:** Jin Zhang, Hao Hong, Chao Lian, Wei Ma, Xiaozhi Xu, Xu Zhou, Huixia Fu, Kaihui Liu, Sheng Meng

**Affiliations:** ^1^ Beijing National Laboratory for Condensed Matter Physics, and Institute of Physics Chinese Academy of Sciences Beijing 100190 P. R. China; ^2^ School of Physical Sciences University of Chinese Academy of Sciences Beijing 100049 P. R. China; ^3^ State Key Laboratory for Mesoscopic Physics School of Physics Peking University Beijing 100871 P. R. China; ^4^ Collaborative Innovation Center of Quantum Matter Beijing 100190 P. R. China

**Keywords:** interlayer‐state‐coupling, MoS_2_/WS_2_ heterostructures, stacking configurations, TDDFT calculations, ultrafast charge transfer

## Abstract

Light‐induced interlayer ultrafast charge transfer in 2D heterostructures provides a new platform for optoelectronic and photovoltaic applications. The charge separation process is generally hypothesized to be dependent on the interlayer stackings and interactions, however, the quantitative characteristic and detailed mechanism remain elusive. Here, a systematical study on the interlayer charge transfer in model MoS_2_/WS_2_ bilayer system with variable stacking configurations by time‐dependent density functional theory methods is demonstrated. The results show that the slight change of interlayer geometry can significantly modulate the charge transfer time from 100 fs to 1 ps scale. Detailed analysis further reveals that the transfer rate in MoS_2_/WS_2_ bilayers is governed by the electronic coupling between specific interlayer states, rather than the interlayer distances, and follows a universal dependence on the state‐coupling strength. The results establish the interlayer stacking as an effective freedom to control ultrafast charge transfer dynamics in 2D heterostructures and facilitate their future applications in optoelectronics and light harvesting.

Since the discovery of graphene and the rise of MoS_2_ as well as black phosphorus, atomically thin 2D crystals have grown into a huge family of materials ranging from semimetal, semiconductors to insulators.^1^, ^2^, ^3^, ^4^, ^5^, ^6^, ^7^, ^8^, ^9^, ^10^, ^11^ Monolayer transition‐metal dichalcogenides denoted as MX_2_ (e.g., M = Mo, W, and X = S, Se, Te), have been prepared by physical exfoliation and chemical vapor deposition, providing more choices for 2D materials. The MX_2_ materials share similar crystalline structures and symmetries, but possess distinct electronic properties in bandgaps, photoabsorption, and spin–orbit coupling strength.^7^, ^8^, ^9^ The heterostructures vertically reassembled from different 2D materials form even richer material systems, and thus provide a new platform for investigating new physics^12^, ^13^, ^14^, ^15^, ^16^ and exploring new applications.^17^, ^18^, ^19^, ^20^, ^21^, ^22^, ^23^, ^24^ The heterostructures of two MX_2_ are of particular interests because many of them form type II heterojunctions,^25^, ^26^, ^27^ which facilitate the efficient separation of photoexcited electrons and holes^28^, ^29^ and therefore exhibit great potentials in the applications of photodetectors,^30^, ^31^ photovoltaic cells,^32^, ^33^ and light emitters.^34^

The interlayer charge transfer in MX_2_ heterostructures is of central importance in their photoresponse, which determines both the speed and efficiency of the charge separation.^35^, ^36^ Since the charge transfer is mainly through the overlapping between interlayer electronic states, the charge transfer process is believed to be highly dependent on the interlayer stackings (twisting, translation, and spacing) and interactions. Femtosecond pump–probe spectroscopy experiments reveals that the excited hole in MoS_2_/WS_2_ bilayer takes place in an ultrafast time scale.^28^ Wang et al. first report that collective motion of excitons at the interface leads to plasma oscillations associated with optical excitation in the ultrafast charge transfer in such van der Waals heterostructures, which provides a good insight in this new pheomenon.^37^ Although both experimental^28^, ^29^ and theoretical efforts^37^, ^38^ have been made to understand the ultrafast charge transfer process in MX_2_ heterostructures, the quantitative characteristic and detailed mechanism for the interlayer stacking/interaction dependence still remain elusive.

Here we utilize the state‐of‐the‐art time‐dependent density functional theory method (TDDFT) and demonstrate a systematical study on the interlayer charge transfer in model MoS_2_/WS_2_ bilayer system with variable stacking configurations. Our results demonstrate that the interlayer twisting, translation, or spacing can significantly modulate interlayer charge transfer time from 100 to 1000 fs scale, a huge modulation that has not been realized before. Further analysis reveals that the transfer rate in MoS_2_/WS_2_ bilayers is governed by the coupling between specific interlayer states (rather than the total coupling strength) and follows a universal exponential dependence on their dipole coupling matrix values. This work establishes a firm correlation between charge transfer dynamics and interlayer stacking/interaction in 2D heterostructures on the atomic level, and thus facilitate their future applications in, for example, high speed optoelectronics and new generations of light harvesting.

The choice of MoS_2_/WS_2_ as a model system to study the interlayer charge transfer process in 2D heterostructures is mainly initiated by the ultrafast experiment from Wang and co‐workers at UC Berkeley.^28^ MoS_2_/WS_2_ bilayers have a type II band alignment: the conduction band minimum and valence band maximum at K point reside at MoS_2_ and WS_2_ layers, respectively [**Figure** **1**; also see Figure S1 in the Supporting Information]. Since MoS_2_ has an energy bandgap smaller than WS_2_, we can selectively excite the MoS_2_ by purposely choosing the excitation light with photon energy in between the bandgap of MoS_2_ and WS_2_. After photoexcitation, holes in MoS_2_ will have the chance to transfer into the WS_2_ because its valence band maximum is lower than that of WS_2_; while the electrons will stay in MoS_2_ since its conduction band minimum is also lower than WS_2_ (Figure 1b).

**Figure 1:**
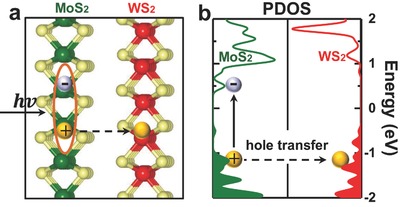
Atomic and electronic structure of the MoS_2_/WS_2_ bilayer. a) Side view of MoS_2_/WS_2_ bilayers. Green, red, and light yellow spheres represent Mo, W, and S atoms, respectively. b) Projected density of states (PDOS) on MoS_2_ and WS_2_ layer. Photoexcited holes will transfer from MoS_2_ valence bands to WS_2_.

MoS_2_/WS_2_ bilayers have many possible interlayer stacking configurations, i.e., AB_1_‐2H and AB_2_‐2H, AA_1_‐3R and AA_2_‐3R, and twisted ones.^39^, ^40^ Here, we choose the most stable AB_1_‐2H as an example to show the time evolution of the interlayer charge transfer. In our calculations, we use Ehrenfest dynamics based on TDDFT approach to study excited‐state dynamics, or ab initio TDDFT‐molecular dynamics (MD) methods,^41^, ^42^ which is widely proven reliable in describing quantum systems, such as optical absorption spectrum in dye‐sensitized TiO_2_ nanowire^43^ and electron injection and electron–hole recombination in dye solar cells.^44^ Optical transition in MoS_2_/WS_2_ bilayer is profoundly determined by the direct excitation of electron–hole pair in the individual layer since the interlayer interactions will only disturb the optical transition slightly.^28^ As shown in the band structure of MoS_2_/WS_2_ bilayer (**Figure** **2**a), at K point in the first Brillouin zone the |−2> and |1> states are mainly distributed on MoS_2_ while |−1> state is on WS_2_. Since the |−2> and |−1> states at K point are located at two different layers (MoS_2_ and WS_2_, respectively), they are referred to as interlayer state. It should be noted that |−2> and |−1> states at K point are not sensitive to interlayer coupling, and they do not change much with the number of layers, while the states at Γ point is quite allergic to the interlayer interactions. We note that direct interlayer excitation has a negligible strength as compared to intralayer excitation due to the minimal wavefunction overlap. Once this occurs, the interlayer exciton is formed right away upon excitation, and no charge transfer takes place in a short time <1 ps. By choosing the photon energy to be that of MoS_2_ bandgap, it will mainly excite the electron from |−2> to |1> state and leaves the holes in MoS_2_ layer. The holes will transfer into WS_2_ layers afterward.

**Figure 2:**
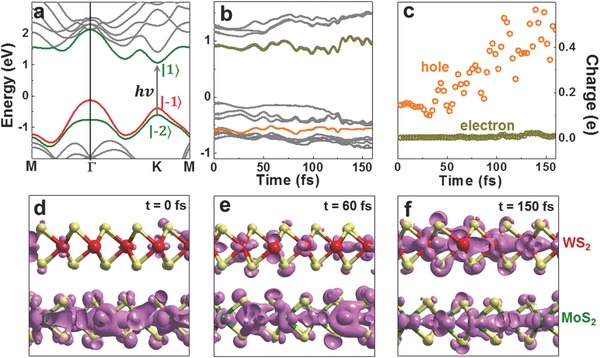
Charge transfer dynamics in AB_1_‐2H stacked MoS_2_/WS_2_ bilayers. a) Energy band dispersion of MoS_2_/WS_2_ bilayer in AB_1_‐2H stacking order. |1>, |−1>, and |−2> indicate these corresponding states at K point in the Brillouin zone. b) Evolution of electronic energy levels after photoexcitation. The orange (dark yellow) curve indicates the energy levels of photoexcited hole (electron) states. c) The fractions of photoexcited holes (electrons) that transferred from MoS_2_ to WS_2_ layer. d–f) Snapshots of the spatial distribution of hole density at 0, 60, and 150 fs after photoexcitation at a contour level of 0.02 e Å^−3^. The upper (lower) layer is WS_2_ (MoS_2_).

In the following, we look into the detailed hole transfer dynamics. We simulate the time evolution of interlayer charge transfer by simultaneously solving the time‐dependent Kohn–Sham equation and the Newtonian motion of ions (ionic forces along the classical trajectory evaluated through the Ehrenfest theorem,^41^, ^42^ see the Experimental Section for details). In Figure 2b, we show the evolution of Kohn–Sham energy levels of MoS_2_/WS_2_ bilayer. At the beginning, the energy of hole states (orange curve) is 0.48 eV below the valence band maximum. As the energy levels evolve in real time accompanied by the ionic motion, the hole states are getting closer to the valence band maximum. At *t* = 150 fs the difference becomes as small as 0.13 eV and the excited hole states in MoS_2_ and original occupied states in WS_2_ get largely mixed, or the hole transfer takes place. In Figure 2d–f, we give out the spatial distribution evolution of the hole states at different time. Immediately after photoexcitation, the holes are mostly distributed in MoS_2_ (<10% of the states are in WS_2_, attributing to the weak interlayer state hybridization). At *t* = 60 fs, ≈25% of the holes have transferred into WS_2_, and clearly at *t* = 150 fs, about 50% of the holes are localized in WS_2_. It means that the hole transfer between MoS_2_ and WS_2_ is taking place in an ultrafast way.

To gain quantitative information on charge transfer dynamics, we integrate the hole (electron) density (χ) on the WS_2_ orbitals at different time after excitation (Figure 2c). Within 150 fs, about half of the holes density has transferred to WS_2_. While as expected the electrons still stay in MoS_2_ and would not transfer to WS_2_ during the whole simulation time. We note that an oscillation of about 30 fs is observed, possibly due to the ultrafast oscillation of electronic states (oscillation of ionic motion is of period much larger than 30 fs and thus can be excluded).^37^ The *A*_1g_ mode was found to be critical to the photoexcited hole dynamics. Here by doing Fourier transformation of dipole moment along the vertical direction of the heterojunction, we find that the *A*_1g_ phonon mode indeed plays an important role in the process of ultrafast hole transfer (see the Supporting Information for details). The formation of intralayer exciton prior to interlayer charge transfer and the collective motion of excitons is secondary for the dynamics of photoexcited charge, because the main driving force in such an ultrafast process is the specific state coupling as detailed below.

Now we turn to evaluate the dependence of charge transfer dynamics on interlayer stackings and interactions. There are five typical stacking configurations of AB_1_‐2H, AB_2_‐2H, AB_3_‐2H, AA_1_‐3R, and AA_2_‐3R in MoS_2_/WS_2_ bilayers. For AB ones, the M‐X bond directions in the two layers are opposite; while for AA ones, the directions are the same. So between AA and AB ones, the bilayers rotate by an angle of π; and between AA_1_‐3R (AB_1_‐2H) and AA_2_‐3R (AB_2_‐2H), the two layers translate in‐plane by 1.1 Å. From our calculations (**Table** **1**) and also previous results,^22^ AB_1_‐2H, AB_2_‐2H, and AA_1_‐3R are the stable configurations with a smaller interlayer spacing of 6.3 Å; while AA_2_‐3R is metastable with a larger interlayer spacing of 6.8 Å. The formation energies of AB_1_‐2H, AB_2_‐2H, and AA_1_‐3R is of similar strength, but much lower than in AA_2_‐3R.^21^ Both AA_2_‐3R and AB_3_‐2H share the similar orientation and interlayer distance (6.8 Å) and hole dynamics. Thus, the hole dynamics of AB_3_‐2H stacking mode is presented for comparison in Figure S6 (Supporting Information). Since the charge transfer is related to the interlayer electronic coupling, naively one would expect that the shorter the interlayer distance is, the faster the interlayer charge transfer will be, or τ_AB1_ ≈ τ_AB2_ ≈ τ_AA1_ ≪ τ_AA2_.

**Table 1:** Calculated parameters for MoS_2_/WS_2_ bilayers

Stacking		*d* a) [Å]	*E* b) [meV per atom]	*M* c) [*e* Å]	τd) [*fs*]
AB_1_‐2H		6.3	−30.6	0.42	150
AB_1_‐2H	Artificial	6.0	−27.5	0.62	120
AB_1_‐2H	Artificial	7.0	−20.0	0.16	320
AB_1_‐2H	Artificial	7.3	−16.2	0.005	1600
AB_2_‐2H		6.3	−28.3	0.06	1100
AA_1_‐3R		6.3	−28.7	0.02	1500
AA_2_‐3R		6.8	−19.7	0.18	180

^a)^ *d*: interlayer spacing

^b)^ *E*: formation energy

^c)^ *M*: dipole transition matrix element; and

^d)^ τ: hole transfer lifetime.

In our results, we fit the time evolution data with an exponential equation χ = a + b*exp (−*t*/τ), where τ is the charge transfer lifetime. As marked in **Figure** **3**a–d, τ_AB1_ or τ_AA2_ is around 100 fs timescale and τ_AB2_ or τ_AA1_ is around 1000 fs scale (or τ_AB1_ ≈ τ_AA2_ ≪ τ_AB2_ ≈ τ_AA1_). It seems that the charger transfer time has no obvious correlation to interlayer coupling strength, which is quite counter‐intuitive. The timescale is obtained by fitting to the simulated trends of ultrafast charge transfer upon photoexcitation. Direct simulation with trajectories longer than picoseconds is not only computationally unaffordable, but also blocked by the divergence of orbital propagation and break‐down of mean field approach. We found that simulation in a relatively short timescale already gives a very reasonable fitting result of the charge transfer timescale (Figure S5, Supporting Information).

**Figure 3:**
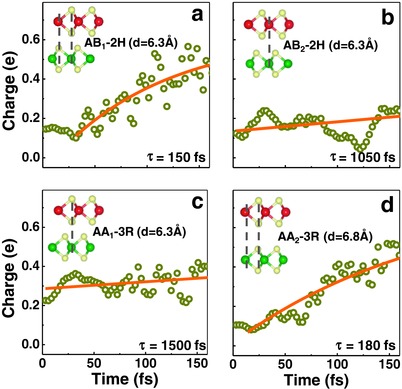
a–d) Hole transfer evolution for MoS_2_/WS_2_ in different stacking configurations. The transfer lifetime is fitted by an exponential relation. The insets give out the schematic atomic structure, interlayer spacing, and lifetime. From the consideration of total interlayer distances, one would expect τ_AB1_ ≈ τ_AB2_ ≈ τ_AA1_ ≪ τ_AA2_, but the simulation results show τ_AB1_ ≈ τ_AA2_ ≪ τ_AB2_ ≈ τ_AA1_. It reveals that the charger transfer time has no obvious correlation to the total interlayer coupling strength.

The above observed discrepancy between computation data and expectation drives us to rethink about the interlay couplings. The usual mechanical or electronic interlayer coupling we talked about is actually the total one, which includes all the electronic states. However, the interlayer charge transfer takes place only between some specific interlayer states, and the charger transfer may be only related to the coupling between these specific ones. We therefore evaluate all the coupling elements between different specific interlayer states and eventually figure out the one that is responsible for the charge transfer dynamics, namely, the one between the |−2> and |−1> states at K point in the Brillouin zone. We use the dipole transition matrix element (*M*) to evaluate the coupling strength between the two states as

(1) $$M=\;<-2|\hat{Z}|-1>,$$

where $\hat{Z}$ is the position operator along the vertical direction normal to the MX_2_ plane. Quite interestingly, we found that *M*_AB1_ > *M*_AA2_ ≫ *M*_AB2_ > *M*_AA1_, which is positively correlated to 1/τ. Detailed analysis further revealed that 1/τ is exponentially dependent on *M*, or 1/τ ∝ *e^M^* (dashed line in **Figure** **4**a).

**Figure 4:**
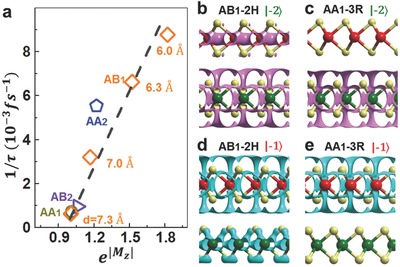
Interlayer‐state‐coupling dependent charge transfer rate in MoS_2_/WS_2_ bilayers. a) Dependence of the charge transfer rates (1/τ) on the dipole transition coupling strength (*M*) between |−2> and |−1> states. AA_1_‐3R, AA_2_‐3R, AB_1_‐2H, and AB_2_‐2H data are, respectively, shown as circle, pentagon, square, and triangles. The interlayer spacing value of AB_1_‐2H (including those artificial ones) are labeled beside the data points. All data seat around the same curve, revealing a universal dependence of charge transfer rate on interlayer‐state‐coupling strength. b–e) Spatial distribution of |−2> and |−1> states in AB_1_‐2H and AA_1_‐3R stackings. The overlapping between |−2> and |−1> states along vertical direction on both the upper WS_2_ and lower MoS_2_ layers in AB_1_‐2H stacking is finite, while it is nearly zero in AA_1_‐3R stacking. Thus *M*_AB1_ ≫ *M*_AA1_ is understandable. The isosurface value is 1 × 10^−3^ e Å^−3^ in (b–e).

In ref. 37, using both TDDFT and model Hamiltonian analysis Wang et al. studied dipole transition couplings between different layers in the heterojunctions and showed charge transfer time is dependent on stacking parameters. In the present work, the first principle simulations were further extended to different systems and longer times, where the charge transfer time is determined for every single stacking mode and thus the exponential dependence of charge transfer time on the interlayer‐state‐coupling strength can be plotted.

To further check whether this exponential dependence is universal in determining the interlayer charge transfer dynamics, we artificially tune the interlayer spacing from 6.0 to 7.3 Å in AB_1_‐2H stacking and obtained the τ and *M* (see Figure S2 in the Supporting Information for details). Again, all the data seat around the same 1/τ versus *e^M^* curve. So, the interlayer coupling between the |−2> and |−1> states is the universal factor that determines the charge transfer dynamics in MoS_2_/WS_2_ bilayers. To clarify why the specific coupling strength between |−2> and |−1> states at K point is crucial, we have checked all relative states (occupied or unoccupied states) and found only $M=\;<-2|\hat{Z}|-1>$, is allergic to the photoexcited hole dynamics (with a typical value of 0.5 e Å for AB_1_‐2H stacking). The couplings between other pairs of states are much smaller (with a typical value of 10^−3^ e⋅ Å). The dependence of charge transfer rate on the interlayer‐state‐coupling *M* justifies the importance of *M* as a measure for interface ultrafast dynamics.

Now we try to give out a physical picture why slight change of interlayer stacking configuration will give out dramatically different *M*. We use AB_1_‐2H and AA_1_‐3R bilayers as an example for illustration, which are both stable with the interlayer spacing of 6.3 Å. We directly draw the spatial distribution of |−2> (Figure 4b) and |−1> states (Figure 4 d) in both WS_2_ and MoS_2_ layers. For AB_1_‐2H stacking, in the upper WS_2_ layer, |−2> has finite overlapping with |−1> states along vertical direction and so as in the lower MoS_2_ layer. In contrast, for AA_1_‐3R stacking, the overlapping in both layers is nearly zero. Since the $\hat{Z}$ is an operation that projects electronic states to vertical directions, therefore *M*_AB1_ ≫ *M*_AA1_ is understandable (see Figure S3 in the Supporting Information for the other two stackings).

In the original experiment by Hong et al., the authors used film transfer method to prepare MoS_2_/WS_2_ bilayers, where the interlayer stacking configurations are not well controlled.^28^ Recently, MX_2_ bilayers with defined interlayer geometry can be directly grown by chemical vapor deposition methods in several groups.^15^, ^20^, ^21^ And to utilize the two‐color pump–probe optical technique, the charge transfer lifetime can be obtained by evaluating the rising up part of the pump–probe curve.^28^, ^29^ Therefore, it is now the right time to experimentally study the charge transfer dynamics in MoS_2_/WS_2_ bilayers with different stackings predicted here.

In summary, we employ ab initio TDDFT‐MD methods to investigate the ultrafast interlayer charge transfer dynamics in MoS_2_/WS_2_ bilayers. Our study reveals that slight interlayer geometry modulation of twisting, translation, or spacing can tune charge transfer dynamics very effectively, resulting in a change of transfer lifetime spanning from 100 to 1000 fs timescale. A universal exponential relationship between the charge transfer rate and the coupling strength between specific interlayer electronic states is established, based on detailed analysis of stacking‐mode and layer‐spacing modulation on the charge transfer dynamic processes. Based on these findings, one could utilize physical or chemical methods to control the interlayer geometry and therefore to control the charge transfer quantum dynamics, thus facilitating future applications of 2D heterostructures in novel optoelectronic and light harvesting devices.

## Experimental Section

First principle calculations of MX_2_ vertical heterostructures were performed using density functional theory implemented in the Vienna ab initio simulation package^45^ with Perdew, Burke, and Ernzerhof (PBE) generalized gradient approximation for the exchange–correlation functional.^46^, ^47^ Because of the absence of strong chemical bonding between layers, van der Waals density functional in the opt88 form^48^ was employed for structural optimization. Both lattice constants and atomic positions were relaxed until all residual forces remain less than 10^−2^ eV Å^−1^ and the total energy variation is less than 10^−4^ eV. The Brillouin zone was sampled by a set of 25 × 25 × 1 k‐mesh with an energy cutoff of 400 eV for plane waves. The thickness of vacuum layer was set to be larger than 15 Å, so that interactions between repeated images are avoided. Formation energy (*E*) of the MoS_2_/WS_2_ heterostructure is defined as $E=(E_{{\mathrm{MoS}}_{2}/{\mathrm{WS}}_{2}}-\;E_{{\mathrm{MoS}}_{2}}-\;E_{{\mathrm{WS}}_{2}})/N$, where $E_{{\mathrm{MoS}}_{2}/{\mathrm{WS}}_{2}}$, $E_{{\mathrm{MoS}}_{2}}$, $E_{{\mathrm{WS}}_{2}}$ are, respectively, the total energies of MoS_2_/WS_2_ bilayers, MoS_2_ and WS_2_ layers, and *N* is the number of atoms in the supercells.

The excited‐state real‐time TDDFT simulations were carried out with the time‐dependent ab initio package TDAP^42^ based on SIESTA.^49^ Pseudopotentials of the Troullier–Martins type, the PBE exchange–correlation functional, and a local basis set of double‐ζ polarized orbitals were used. It was noted that the band offset between the valence band maximum of WS_2_ and MoS_2_ in experiment (0.7 eV) were correctly reproduced by the PBE functional (0.5 eV).^50^ Although PBE functional usually underestimates the bandgaps, it is accurate enough to describe the spatial distribution of electronic states and the state couplings, which are crucial in the dynamic simulations.^51^ In addition, very similar band structures based on PBE functional and HSE06 functional are shown in Figure S7 (see the Supporting Information for details). Supercells in rectangle shape containing 108 atoms in the supercells were used to model bilayers with a single k‐point for integration in the Brillouin zone and the states relative to the photoexcited states especially at K point in the first Brillouin zone were approximately folded to the supercells. The time step of all simulations was set to be 0.024 fs. Electron–hole interaction and electron–phonon effects were naturally included in the methods. The initial velocities of ions were assigned according to the equilibrium Boltzmann–Maxwell distribution at a given temperature of 350 K. It should be noted that some energy levels in the supercells were used may be degenerate, so states with similar energy and spatial distributions have been checked in the following dynamic simulations to ensure the reliability of the results.

## Conflict of Interest

The authors declare no conflict of interest.

## Supporting information

Supplementary

## References

[1] A. K. Geim , K. S. Novoselov , Nat. Mater. 2007, 6, 183. 17330084 10.1038/nmat1849

[2] A. Frenzel , C. Lui , Y. Shin , J. Kong , N. Gedik , Phys. Rev. Lett. 2014, 113, 056602. 25126929 10.1103/PhysRevLett.113.056602

[3] A. Splendiani , L. Sun , Y. Zhang , T. Li , J. Kim , C.‐Y. Chim , G. Galli , F. Wang , Nano Lett. 2010, 10, 1271. 20229981 10.1021/nl903868w

[4] K. F. Mak , C. Lee , J. Hone , J. Shan , T. F. Heinz , Phys. Rev. Lett. 2010, 105, 136805. 21230799 10.1103/PhysRevLett.105.136805

[5] K. F. Mak , K. L. McGill , J. Park , P. L. McEuen , Science 2014, 344, 1489. 24970080 10.1126/science.1250140

[6] Z. Ye , T. Cao , K. O'Brien , H. Zhu , X. Yin , Y. Wang , S. G. Louie , X. Zhang , Nature 2014, 513, 214. 25162523 10.1038/nature13734

[7] Q. H. Wang , K. Kalantar‐Zadeh , A. Kis , J. N. Coleman , M. S. Strano , Nat. Nanotechnol. 2012, 7, 699. 23132225 10.1038/nnano.2012.193

[8] F. Xia , H. Wang , D. Xiao , M. Dubey , A. Ramasubramaniam , Nat. Photonics 2014, 8, 899.

[9] B. W. Baugher , H. O. Churchill , Y. Yang , P. Jarillo‐Herrero , Nat. Nanotechnol. 2014, 9, 262. 24608231 10.1038/nnano.2014.25

[10] H. Wang , C. Zhang , F. Rana , Nano Lett. 2015, 15, 339. 25546602 10.1021/nl503636c

[11] L. Li , Y. Yu , G. J. Ye , Q. Ge , X. Ou , H. Wu , D. Feng , X. H. Chen , Y. Zhang , Nat. Nanotechnol. 2014, 9, 372. 24584274 10.1038/nnano.2014.35

[12] A. K. Geim , I. V. Grigorieva , Nature 2013, 499, 419. 23887427 10.1038/nature12385

[13] B. Hunt , J. D. Sanchez‐Yamagishi , A. F. Young , M. Yankowitz , B. J. LeRoy , K. Watanabe , T. Taniguchi , P. Moon , M. Koshino , P. Jarillo‐Herrero , Science 2013, 340, 1427. 23686343 10.1126/science.1237240

[14] C. H. Lee , G. H. Lee , A. M. Van Der Zande , W. Chen , Y. Li , M. Han , M. X. Cui , G. Arefe , C. Nuckolls , T. F. Heinz , Nat. Nanotechnol. 2014, 9, 676. 25108809 10.1038/nnano.2014.150

[15] Y. Gong , J. Lin , X. Wang , G. Shi , S. Lei , Z. Lin , X. Zou , G. Ye , R. Vajtai , B. I. Yakobson , Nat. Mater. 2014, 13, 1135. 25262094 10.1038/nmat4091

[16] M.‐H. Chiu , C. Zhang , H.‐W. Shiu , C.‐P. Chuu , C.‐H. Chen , C.‐Y. S. Chang , C.‐H. Chen , M.‐Y. Chou , C.‐K. Shih , L.‐J. Li , Nat. Commun. 2015, 6, 7666. 26179885 10.1038/ncomms8666 PMC4518320

[17] H. Heo , J. H. Sung , S. Cha , B.‐G. Jang , J.‐Y. Kim , G. Jin , D. Lee , J.‐H. Ahn , M.‐J. Lee , J. H. Shim , Nat. Commun. 2015, 6, 7372. 26099952 10.1038/ncomms8372 PMC4557351

[18] A. F. Rigosi , H. M. Hill , Y. Li , A. Chernikov , T. F. Heinz , Nano Lett. 2015, 15, 5033. 26186085 10.1021/acs.nanolett.5b01055

[19] P. Rivera , J. R. Schaibley , A. M. Jones , J. S. Ross , S. Wu , G. Aivazian , P. Klement , K. Seyler , G. Clark , N. J. Ghimire , Nat. Commun. 2015, 6, 6242. 25708612 10.1038/ncomms7242

[20] Q. Zhang , X. Xiao , R. Zhao , D. Lv , G. Xu , Z. Lu , L. Sun , S. Lin , X. Gao , J. Zhou , Angew. Chem., Int. Ed. 2015, 54, 8957. 10.1002/anie.201502461 26118436

[21] J. Zhang , J. Wang , P. Chen , Y. Sun , S. Wu , Z. Jia , X. Lu , H. Yu , W. Chen , J. Zhu , Adv. Mater. 2016, 28, 1950. 26708256 10.1002/adma.201504631

[22] S. Han , H. Kwon , S. K. Kim , S. Ryu , W. S. Yun , D. Kim , J. Hwang , J.‐S. Kang , J. Baik , H. Shin , Phys. Rev. B 2011, 84, 045409.

[23] L. Zhang , A. Zunger , Nano Lett. 2015, 15, 949. 25562378 10.1021/nl503717p

[24] J. Kang , L. Zhang , S. H. Wei , J. Phys. Chem. Lett. 2016, 7, 597. 26800573 10.1021/acs.jpclett.5b02687

[25] D. Meier , T. Kuschel , L. Shen , A. Gupta , T. Kikkawa , K.‐I. Uchida , E. Saitoh , J.‐M. Schmalhorst , G. Reiss , Phys. Rev. B 2013, 87, 054421.

[26] J. Kang , S. Tongay , J. Zhou , J. Li , J. Wu , Appl. Phys. Lett. 2013, 102, 012111.

[27] C. Gong , H. Zhang , W. Wang , L. Colombo , R. M. Wallace , K. Cho , Appl. Phys. Lett. 2013, 103, 053513.

[28] X. Hong , J. Kim , S.‐F. Shi , Y. Zhang , C. Jin , Y. Sun , S. Tongay , J. Wu , Y. Zhang , F. Wang , Nat. Nanotechnol. 2014, 9, 682. 25150718 10.1038/nnano.2014.167

[29] F. Ceballos , M. Z. Bellus , H.‐Y. Chiu , H. Zhao , ACS Nano 2014, 8, 12717. 25402669 10.1021/nn505736z

[30] L. Britnell , R. Ribeiro , A. Eckmann , R. Jalil , B. Belle , A. Mishchenko , Y.‐J. Kim , R. Gorbachev , T. Georgiou , S. Morozov , Science 2013, 340, 1311. 23641062 10.1126/science.1235547

[31] W. J. Yu , Y. Liu , H. Zhou , A. Yin , Z. Li , Y. Huang , X. Duan , Nat. Nanotechnol. 2013, 8, 952. 24162001 10.1038/nnano.2013.219 PMC4249654

[32] M. Bernardi , M. Palummo , J. C. Grossman , Nano Lett. 2013, 13, 3664. 23750910 10.1021/nl401544y

[33] Y. Yu , S. Hu , L. Su , L. Huang , Y. Liu , Z. Jin , A. A. Purezky , D. B. Geohegan , K. W. Kim , Y. Zhang , Nano Lett. 2014, 15, 486. 25469768 10.1021/nl5038177

[34] J. S. Ross , P. Klement , A. M. Jones , N. J. Ghimire , J. Yan , D. Mandrus , T. Taniguchi , K. Watanabe , K. Kitamura , W. Yao , Nat. Nanotechnol. 2014, 9, 268. 24608230 10.1038/nnano.2014.26

[35] S. M. Falke , C. A. Rozzi , D. Brida , M. Maiuri , M. Amato , E. Sommer , A. De Sio , A. Rubio , G. Cerullo , E. Molinari , Science 2014, 344, 1001. 24876491 10.1126/science.1249771

[36] C. A. Rozzi , S. M. Falke , N. Spallanzani , A. Rubio , E. Molinari , D. Brida , M. Maiuri , G. Cerullo , H. Schramm , J. Christoffers , Nat. Commun. 2013, 4, 1602. 23511467 10.1038/ncomms2603 PMC3615481

[37] H. Wang , J. Bang , Y. Sun , L. Liang , D. West , V. Meunier , S. Zhang , Nat. Commun. 2016, 7, 11504. 27160484 10.1038/ncomms11504 PMC4866324

[38] R. Long , O. V. Prezhdo , Nano Lett. 2016, 16, 1996. 26882202 10.1021/acs.nanolett.5b05264

[39] A. M. van Der Zande , J. Kunstmann , A. Chernikov , D. A. Chenet , Y. You , X. Zhang , P. Y. Huang , T. C. Berkelbach , L. Wang , F. Zhang , Nano Lett. 2014, 14, 3869. 24933687 10.1021/nl501077m

[40] K. Liu , L. Zhang , T. Cao , C. Jin , D. Qiu , Q. Zhou , A. Zettl , P. Yang , S. G. Louie , F. Wang , Nat. Commun. 2014, 5, 4966. 25233054 10.1038/ncomms5966

[41] E. Runge , E. K. Gross , Phys. Rev. Lett. 1984, 52, 997.

[42] S. Meng , E. Kaxiras , J. Chem. Phys. 2008, 129, 054110. 18698891 10.1063/1.2960628

[43] S. Meng , J. Ren , E. Kaxiras , Nano Lett. 2008, 8, 3266. 18788788 10.1021/nl801644d

[44] S. Meng , E. Kaxiras , Nano Lett. 2010, 10, 1238. 20353199 10.1021/nl100442e

[45] G. Kresse , J. Furthmüller , Comput. Mater. Sci. 1996, 6, 15.

[46] P. E. Blöchl , Phys. Rev. B 1994, 50, 17953. 10.1103/physrevb.50.17953 9976227

[47] J. P. Perdew , K. Burke , M. Ernzerhof , Phys. Rev. Lett. 1996, 77, 3865. 10062328 10.1103/PhysRevLett.77.3865

[48] J. Klimeš , D. R. Bowler , A. Michaelides , Phys. Rev. B 2011, 83, 195131.

[49] J. M. Soler , E. Artacho , J. D. Gale , A. García , J. Junquera , P. Ordejón , D. J. Sánchez‐Portal , J. Phys.: Condens. Matter. 2002, 14, 2745. 10.1088/0953-8984/20/6/064208 21693870

[50] H. M. Hill , A. F. Rigosi , K. T. Rim , G. W. Flynn , T. F. Heinz , Nano Lett. 2016, 16, 4831. 27298270 10.1021/acs.nanolett.6b01007

[51] W. Ma , J. Zhang , L. Yan , Y. Jiao , Y. Gao , S. Meng , Comput. Mater. Sci. 2016, 112, 478.

