# Supplementary material for: Interlayer‐State‐Coupling Dependent Ultrafast Charge Transfer in MoS2/WS2 Bilayers
Source: Adv Sci (Weinh). 2017 Apr 24;4(9):1700086. doi: 10.1002/advs.201700086 (PMC5604380; doi:10.1002/advs.201700086)
Supplement: Supplementary file 1 — Supplementary [file ADVS-4-na-s001.pdf]

Copyright WILEY-VCH Verlag GmbH & Co. KGaA, 69469 Weinheim, Germany, 2017.

Supporting Information

for *Adv. Sci.,* DOI: 10.1002/advs.201700086

Interlayer-State-Coupling Dependent Ultrafast Charge Transfer in MoS_2/WS_2 Bilayers

Jin Zhang, Hao Hong, Chao Lian, Wei Ma, Xiaozhi Xu, Xu Zhou, Huixia Fu, Kaihui Liu, and Sheng Meng**

Supporting Information for

Interlayer-State-Coupling Dependent Ultrafast Charge Transfer in MoS_2/WS_2 Bilayers

Jin Zhang[1,4⊥], Hao Hong[2⊥], Chao Lian[1⊥], Wei Ma[1,4], Xiaozhi Xu[2], Xu Zhou[2], Huixia Fu[1,4], Kaihui Liu[2,3*], and Sheng Meng[1,3,4*]

[1]Beijing National Laboratory for Condensed Matter Physics, and Institute of Physics, Chinese Academy of Sciences, Beijing 100190, P. R. China

[2]State Key Laboratory for Mesoscopic Physics, School of Physics, Peking University, Beijing 100871, P. R. China

[3]Collaborative Innovation Center of Quantum Matter, Beijing 100190, P. R. China

[4]School of Physical Science, University of Chinese Academy of Sciences, Beijing 100049, P. R. China

This file contains:

S1. Atomic structures and energy bands of MoS_2/WS_2 bilayers

S2. Interlayer spacing modulated hole transfer dynamics in MoS_2/WS_2 bilayers

S3. Spatial dispersion of |-1> and |-2> states in MoS_2/WS_2 bilayers

S4. Fourier transformation of dipole moments

S5, Longer evolution of excited hole state in AB_1-2H stacking order

S6, Hole transfer dynamics for MoS_2/WS_2 in AB_3-2H stacking order

S7, Band structures of MoS_2/WS_2 in HSE06 functional

S1. Atomic structures and energy bands of MoS_2/WS_2 bilayers

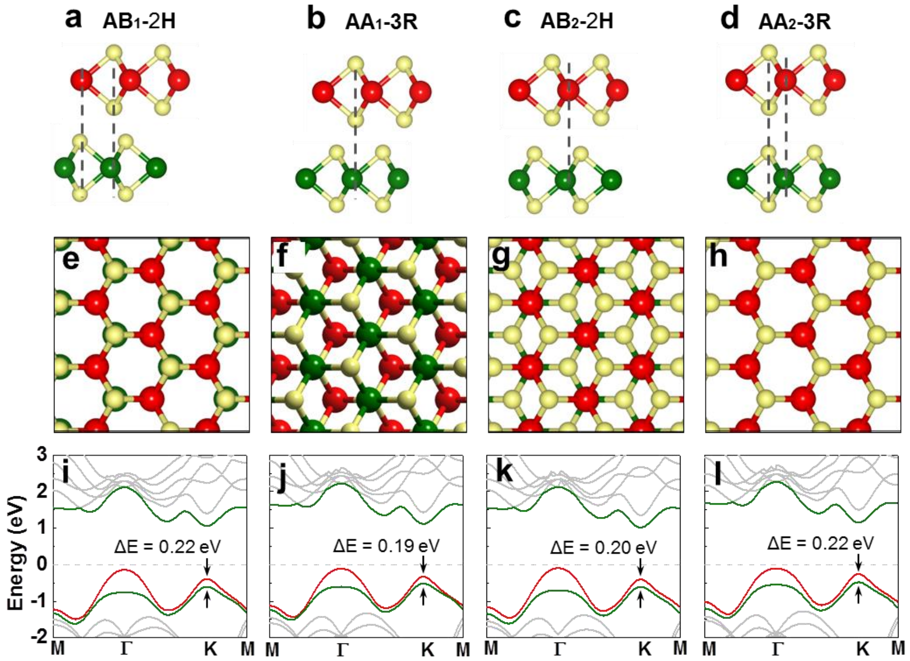

Figure S1. Schematic atomic structures and energy bands of MoS_2/WS_2 bilayers with different stackings. (a-d) Top views of different stacking configurations. In AB_1-2H stacking, Mo (S) atoms in MoS_2 layer is located right above the S (W) atoms of WS_2 layer. In AA_2 stacking, Mo (S) atoms in MoS_2 layer is exactly on W (S) atoms in WS_2 layer. By shifting AB_1-2H (AA_2-3R) stacking along armchair direction by one in-plane M-S bond length, we obtain AB_1-2H (AA_2-3R)stacking; (e-h) Energy bands for the four different stacking configurations and energy differences between |-2> and |-1> states at K point in the Brillouin zone are also given. The very small energy difference reveals that the driving forces (energy difference between |-2> and |-1> states) is not the reason for the difference in excited charge dynamics.

S2. Interlayer spacing modulated hole transfer dynamics in MoS_2/WS_2 bilayers

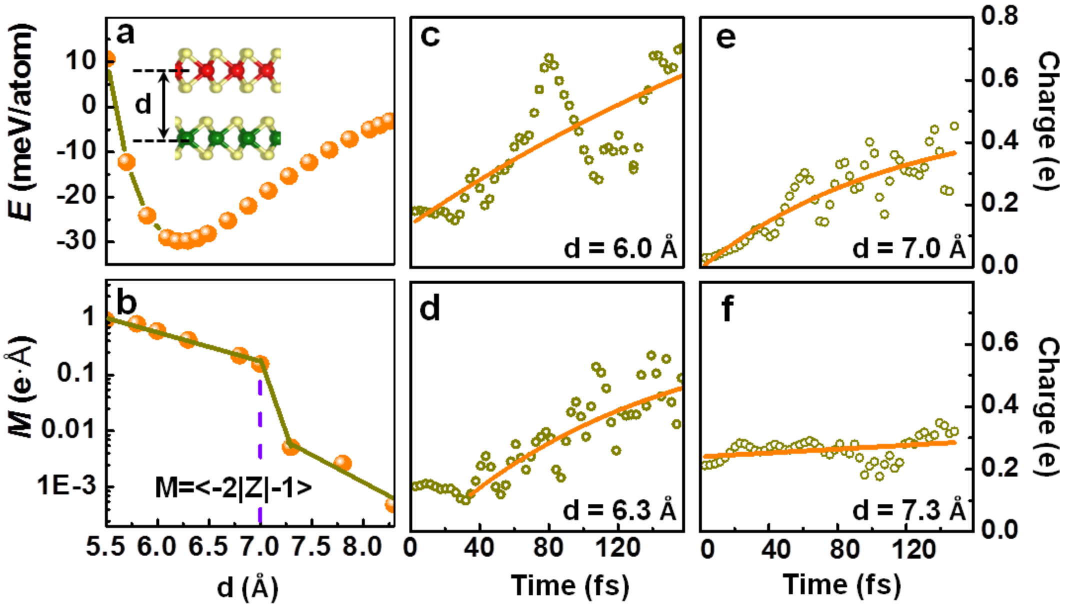

Figure S2. Interlayer spacing modulated hole transfer dynamics for AB_1 stacking MoS_2/WS_2 bilayers. (a) Variation of the formation energy (*E*) with the interlayer spacing (*d*). (b) Variation of dipole transition matrix element (*M*) with the interlayer spacing. (c-f) The hole transfer dynamic curve under different interlayer spacing.

S3. Spatial dispersion of |-2> and |-2> states in MoS_2/WS_2 bilayers

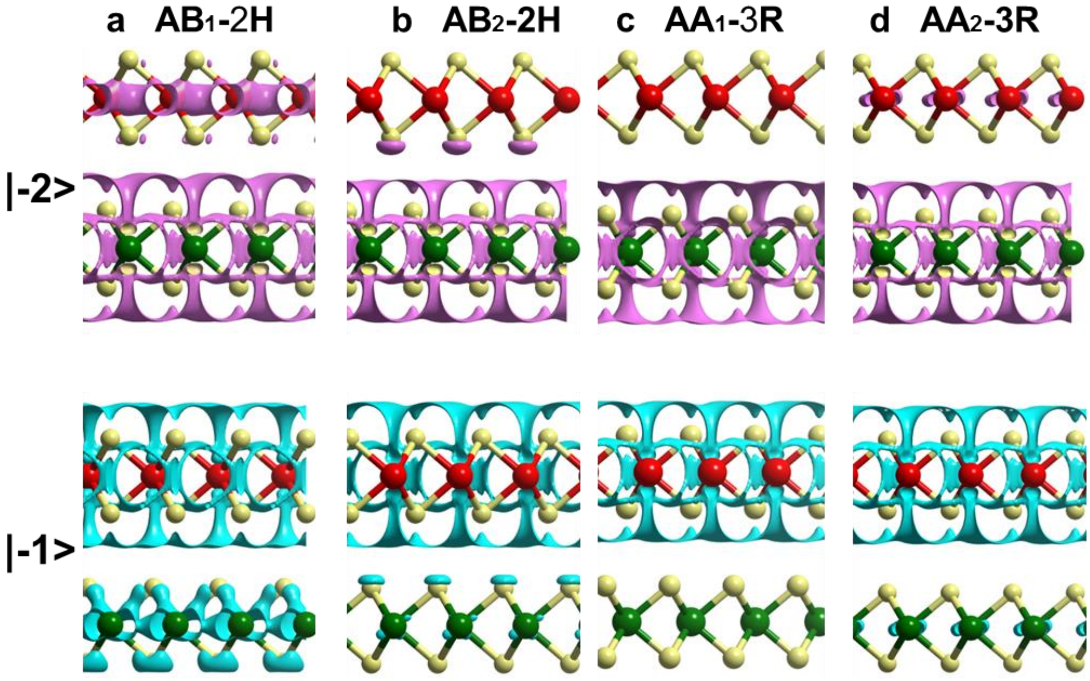

Figure S3. Spatial dispersion of state |-2> and state |-1> of MoS_2/WS_2 bilayers in the four typical stacking modes. The upper layer is MoS_2 and the lower layer is WS_2. The contour level is of 1×10^{-3} e/Å^3.

S4. Fourier transformation of dipole moments

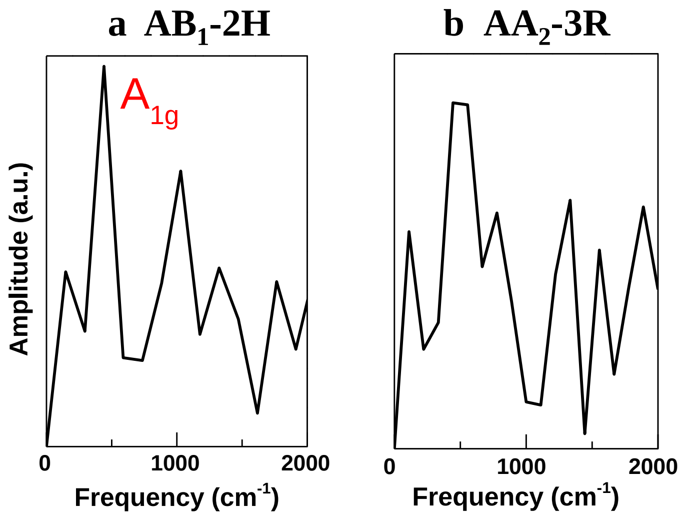

Figure S4. Fourier transformation of the evolution of dipole moments along the direction vertical to MoS_2 and WS_2 layers, illustrating that A_{1g} modes (~400 cm^{-1}) of the heterostructure play an important role in the photoexcited hole dynamics in MoS_2/WS_2 bilayers.

S5, Longer evolution of excited hole state in AB_1-2H stacking order

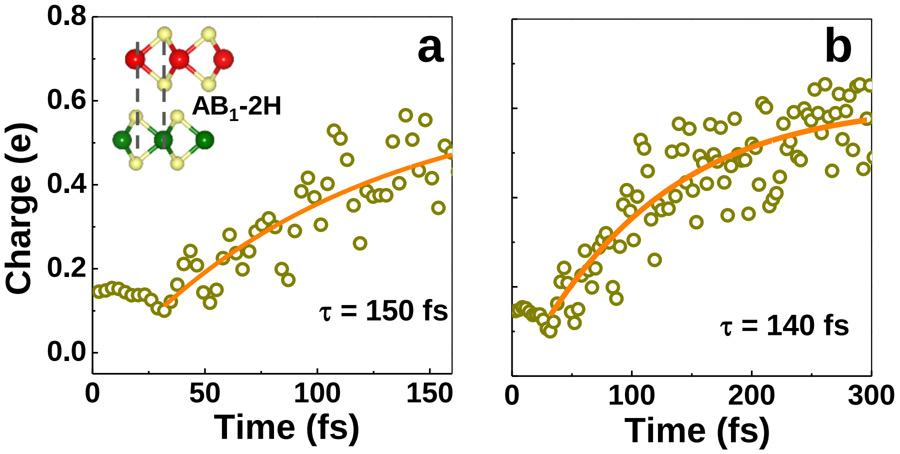

Figure S5. Original (a) and longer (b) evolution of excited hole state in AB_1-2H stacking order to test the accuracy of the exponential fitting of hole dynamics. We can see a longer simulation confirm the reliability of exponential fitting of hole dynamics. We find that simulation in a relatively short timescale (compared with a twice-longer simulation) already gives a very reasonable fitting result (150 fs vs. 140 fs) of the charge transfer time.

S6, Hole transfer dynamics for MoS_2/WS_2 in AB_3-2H stacking order

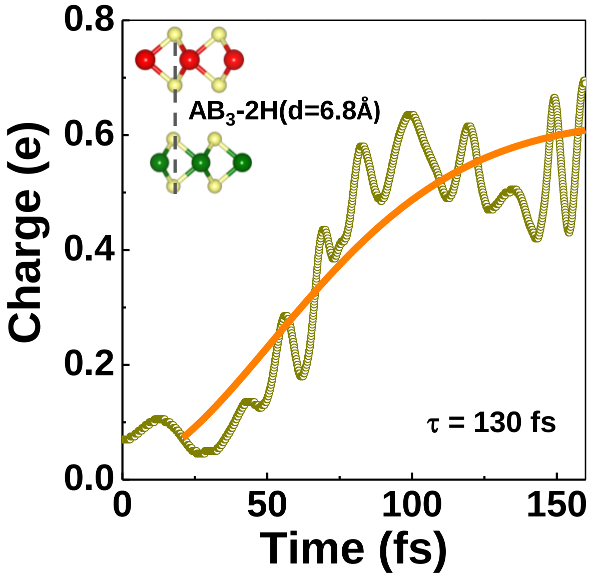

Figure S6. Hole transfer dynamics for MoS_2/WS_2 in AB_3-2H stacking. The timescale of hole transfer from an exponential fitting is 130 fs for this stacking mode.

To clarify the question more clearly, we also calculate the band structures based on HSE06 which is considered to obtain better band alignments. The shape and band offsets from PBE are good enough compared with that from HSE06 functional.

S7, Band structures of MoS_2/WS_2 in HSE06 functional

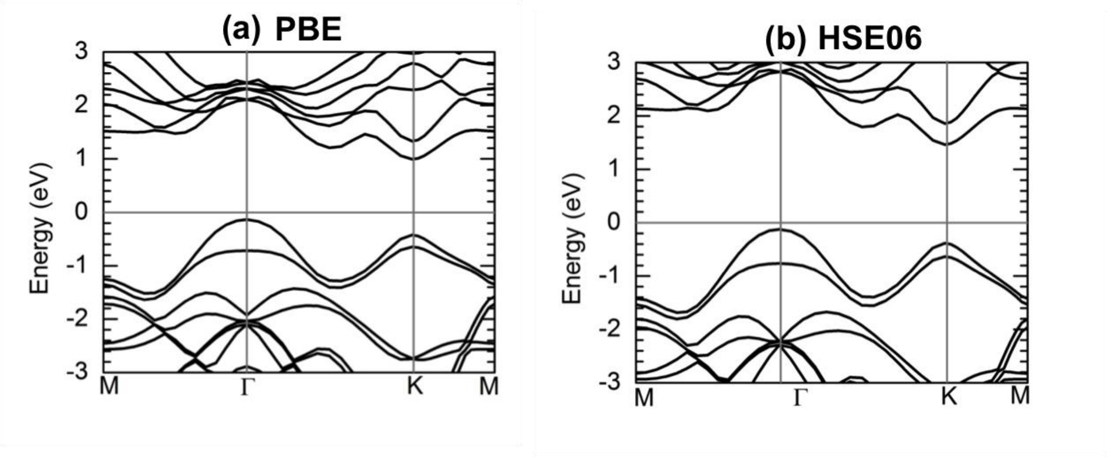

Figure S7. Band structures of MoS_2/WS_2 bilayer in AB-2H stacking calculated using PBE (a) and HSE06 functional (b), respectively. It is apparent that the shape and relative band energies of conduction bands at PBE level are in good consistence with HSE06 calculations. Therefore, PBE functional is good enough to describe photoexcited charge dynamics in MoS_2/WS_2 bilayers.
